# Expression of BARD1 β Isoform in Selected Pediatric Tumors

**DOI:** 10.3390/genes12020168

**Published:** 2021-01-26

**Authors:** Anna Jasiak, Natalia Krawczyńska, Mariola Iliszko, Katarzyna Czarnota, Kamil Buczkowski, Joanna Stefanowicz, Elżbieta Adamkiewicz-Drożyńska, Grzegorz Cichosz, Ewa Iżycka-Świeszewska

**Affiliations:** 1 Department of Biology and Medical Genetics, Medical University of Gdansk, 1 Debinki St., 80-211 Gdansk, Poland; anna.jasiak@gumed.edu.pl (A.J.); milisz@gumed.edu.pl (M.I.); grzcichosz@gmail.com (G.C.); 2 Laboratory of Clinical Genetics, University Clinical Centre, 17 Smoluchowskiego St., 80-210 Gdansk, Poland; 3 Department of Molecular and Integrative Physiology, University of Illinois at Urbana-Champaign, 407S Goodwin Ave, Urbana, IL 61801, USA; natalia.krawczynska@gumed.edu.pl; 4 Department of Pathology and Neuropathology, Medical University of Gdansk, 1 Debinki St., 80-211 Gdansk, Poland; kat.czarnota@gumed.edu.pl (K.C.); kam.buczkowski92@gmail.com (K.B.); 5 Department of Pathomorphology, Copernicus Hospitals, 1-6 Nowe Ogrody St., 80-803 Gdansk, Poland; 6 Department of Pediatrics, Hematology, Oncology, Medical University of Gdansk, 1 Debinki St., 80-211 Gdansk, Poland; joanna.stefanowicz@gumed.edu.pl (J.S.); elzbieta.adamkiewicz-drozynska@gumed.edu.pl (E.A.-D.)

**Keywords:** BARD1, BARD1 β, BARD1 isoforms, splicing, pediatric tumor, germ cell tumors, yolk sac tumors, rhabdomyosarcoma, neuroblastoma, TERT

## Abstract

Currently, many new possible biomarkers and mechanisms are being searched and tested to analyse pathobiology of pediatric tumours for the development of new treatments. One such candidate molecular factor is *BARD1* (BRCA1 Associated RING Domain 1)—a tumour-suppressing gene involved in cell cycle control and genome stability, engaged in several types of adult-type tumours. The data on *BARD1* significance in childhood cancer is limited. This study determines the expression level of *BARD1* and its isoform beta (β) in three different histogenetic groups of pediatric cancer—neuroblastic tumours, and for the first time in chosen germ cell tumours (GCT), and rhabdomyosarcoma (RMS), using the qPCR method. We found higher expression of beta isoform in tumour compared to healthy tissue with no such changes concerning *BARD1* full-length. Additionally, differences in expression of *BARD1* β between histological types of neuroblastic tumours were observed, with higher levels in ganglioneuroblastoma and ganglioneuroma. Furthermore, a higher expression of *BARD1* β characterized yolk sac tumours (GCT type) and RMS when comparing with non-neoplastic tissue. These tumours also showed a high expression of the *TERT* (Telomerase Reverse Transcriptase) gene. In two RMS cases we found deep decrease of BARD1 β in post-chemotherapy samples. This work supports the oncogenicity of the beta isoform in pediatric tumours, as well as demonstrates the differences in its expression depending on the histological type of neoplasm, and the level of maturation in neuroblastic tumours.

## 1. Introduction

**Figure 1:**
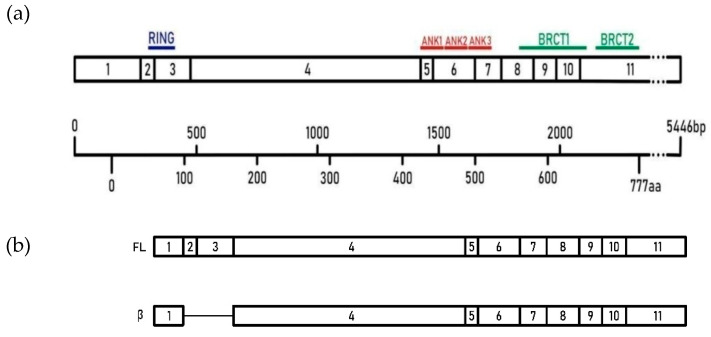
(**a**). Structure of *BARD1*. Exon structure, length, and domain composition of *BARD1 FL* (NCBI Reference Sequence: NM_000465.4, UniProtKB-Q99728). (**b**). Exon structure of full-length *BARD1* and its beta isoform.

Significant patho-clinical heterogeneity of pediatric tumours, together with their low incidence, results in the limited number of ongoing studies, compared to the adult malignancies. Notably, the pathogenesis of pediatric cancers’ differs significantly from adulthood tumours, with a little importance of environmental factors [1]. Cancer is the second cause of death in children, and the reason for morbidity is that most survivors suffer from various long-term side post-treatment effects. Pediatric cancer occurs in the context of development and developmental biology. The genetic predisposition accounts for only 5–10% of pediatric tumours, suggesting other mechanisms, including acquired genetic alterations [2,3]. Alternative RNA splicing, resulting in expression of alternative isoforms, often playing antagonistic functions to the canonical gene product is one of the primary mechanisms in cancer development [4]. BARD1 (BRCA1 Associated RING Domain 1, 2q34-q35) is a leading partner of BRCA1 and consists of 11 exons spanning 777 amino acid protein. *BARD1* has an N-terminal RING-finger domain, three centrally located ankyrin repeats (ANK), and two C-terminal BRCT (BRCA1 C Terminus) domains (Figure 1a) [5,6,7]. A heterodimer of BRCA1 and BARD1 has E3 ubiquitin ligase activity participating in DNA repair, modulating chromatin structure, cell cycle regulation, hormone signaling, as well as some developmental processes [7,8,9]. The function of the BARD1 protein is to form a heterodimer in conjunction with BRCA1 protein. Stable formation of the heterodimer may be critical for BRCA1 exerting cancer inhibition. Full length (FL) *BARD1* is a tumour-suppressor gene playing an essential role in cell cycle control and genome stability. However, *BARD1* encodes at least 11 different isoforms playing antagonistic functions towards FL protein [6,10]. Overexpression of those oncogenic isoforms might result in disrupting the cell cycle and telomeric instability [11]. Mainly, *BARD1* isoforms that lack RING or/and ANK domains were described as oncogenic. Up-regulation of β, δ, ω is associated with unfavorable prognosis in cancers and can antagonize the functions of*BARD1 FL* as a tumour suppressor. Several scientific evidence shows that cancer-associated *BARD1* isoforms act as a driving force for carcinogenesis [6]. *BARD1* β (exon structure shows on Figure 1b), which during mitosis, binds, and stabilizes the Aurora A and B kinases and is overexpressed in several adulthood malignancies [7]. The expression of *BARD1* isoforms was widely studied in several adult type malignancies, such as colorectal, lung, and gynaecological cancers, whereas its role in pediatric tumours is yet to be discovered [12,13,14]. The best-analyzed is neuroblastoma, but only limited data on *BARD1* is available for Wilms’ tumour, case studies in Ewing sarcoma, and osteosarcoma or does not exist as in case of GCT or RMS [15,16,17].

Neuroblastoma is the second most common pediatric solid tumour type growing in the adrenal glands and the abdominal ganglia, mediastinum, and head and neck area [18]. Histologically three categories are discerned: neuroblastoma with undifferentiated, poorly differentiated, and differentiating Schwannian stroma-poor subtypes, ganglioneuroblastoma rich of Schwannian stroma, and ganglioneuroma showing mature phenotype and Schwannian stroma predominance. NB course can be various, but metastatic disease has poor long-term prognosis. The risk group systems aimed for treatment optimalization, are based on patients’ age, tumour location, histology, stage, and molecular changes such as *N-myc* status, chromosomal and single gene abnormalities (ALK, Aurora K) [19,20,21]. Several SNPs in *BARD1* are found to be related to invasive NB. A study comparing 397 high-risk cases and 2043 controls revealed six new SNPs at 2q35 within the *BARD1* gene locus significantly associated with NB. They showed that common variation in *BARD1* associates with the risk of the aggressive and most clinically corresponding subtype of human NB. One of these SNPs has been associated with higher expression of the *BARD1* β isoform [5]. Furthermore, Pugh et al. tested tumour tissue DNA and the matching peripheral blood DNA by using the next sequencing, discovering that *BARD1* could undergo germline mutations [22].

Germ cell tumours (GCT) create a heterogeneous group with various directions and stages of differentiation [23]. Histologically they include two categories–seminomatous (exact names depend on location) and non-seminomatous- encompassing yolk sac tumour (YST), embryonal carcinoma, choriocarcinoma, teratomas, and mixed GCT [24,25,26]. The patho-clinical features, treatment and outcome of GCT are age, histology, and site-dependent. Theories on GCT origin encompass disturbed mitosis-meiosis switch, deregulation of apoptosis and signalling pathways (bFGF, cKIT or SHH), disorganized migration of germ cell precursors and gonadal dysgenesis [27,28]. The molecular pathogenesis of pediatric GCT is not well understood. The genomic profiles in pediatric GCT depend on their location and patient’s age (pre *vs* post-pubertal) [29]. The single gene defects like point mutations are rare and involve mainly *KIT*, *KRAS*, *TP53*, *CDKN2A* [30,31]. Some genes polymorphisms and the biological role of the methylation profiles is also under the investigation in GCTs [32]. To our best knowledge, the role of *BARD1* in GCT has not been investigated so far.

Rhabdomyosarcoma (RMS) is the most common aggressive soft tissue pediatric sarcoma with high metastatic potential and local regrowth [33]. Two principal histological RMS subtypes exist: embryonal and alveolar. The crucial prognostic markers include tumour histology, location, stage and age of the patient. Genetic alterations comprise reciprocal translocations and their associated fusions in alveolar RMS subtype (*PAX, FOXO*), while chromosomal losses and gains in embryonal RMS. The most frequent alterations in RMS include*TP53, NF1, NRAS, MDM2*, and *CDKN2A* aberrations. [34,35]. We have not come across studies on the *BARD1* gene and its isoforms in RMS in the literature so far.

The present study aimed to assess the expression of *BARD1 FL* and *BARD1* β indifferent pediatric tumours. The neuroblastic tumours, chosen germ cell tumours, and a pilot series of rhabdomyosarcoma cases were evaluated to look for possible BARD role in their pathogenesis and clinical characteristics.

## 2. Materials and Methods

Samples (formalin-fixed paraffin-embedded (FFPE) material) included in the study were collected at the time of diagnosis, with all the individuals enrolled being children under 18 years. All cases were clinically and pathologically characterized during previously managed projects (N401 176 31/3867 2005 and NCN 2014/15/B/NZ4/04855). Histological review was performed on hematoxylin-eosin stained preparations. The local ethics committee approved the study at the Medical University of Gdansk. Table 1 contains summarized characteristic of a cohort of neuroblastoma patients.

In total, we analyzed 101 neuroblastoma samples from the patients in the age between 1 month and 14 years. The next examined group consisted of 26 cases of GCTs, where for 21 samples, both tumour and adjacent normal tissue were available. The cohort included seven immature teratomas, nine yolk sac tumours, and ten dysgerminomas. The last group was composed of seven rhabdomyosarcoma (RMS) cases, where for all of them, both tumour and adjacent normal tissue were available. Table 2 and Table 3 show the patho-clinical characteristic of GCTs and RMS group. For three neuroblastoma and two RMS cases tumour tissue was available before and after chemotherapy treatment.

The study material was RNA extracted from formalin-fixed paraffin-embedded tissue (FFPE) using the FormaPure Total (Beckman Coulter, Brea, CA, USA). RNA quality and concentration were assessed using NanoDrop and Qubit, respectively.MultiScribe (Applied Biosystem, Foster City, CA, USA) using random primers was applied for cDNA synthesis, using 200 ng of RNA. Before further analysis, the quality of cDNA was evaluated by amplifying ACTB (Frw: 5′-ATTGGCAATGAGCGGTTC-3’; Rev: 5’CGTGGATGCCACAGGACT -3’), using GoTaq (Promega, Madison, WI, USA) polymerase followed by gel electrophoresis.

A total of 156 samples were qualified for *BARD1* analysis, including97 neuroblastoma samples and 59 samples from 32 patients with other tumours.

Expression analysis for samples was performed using the *BARD1 FL* assay (ID: Hs00957649_m1), spanning exons 3 and 4, while expression of *BARD1* β was evaluated using commercial TaqMan probes spanning exon 1 and 4 (Assay ID: Hs04408502_m1). Additionally, in GCTs and RMS we evaluated expression of TERT, using a specific FAM TaqMan™ Gene Expression Assay (Hs00972650_m1, Thermo Fisher Scientific, Waltham, MA, USA). All the reactions were run in triplicates. The analysis was performed on a LightCycler^®^ 480 Real-Time PCR System (Roche, Basel, Switzerland). Target amplicons were assessed against a control sequence of two reference genes: *PGK1* (Assay ID: Hs99999906_m1) and *IPO8* (Assay ID: Hs00183533_m1). The amplification curves were analyzed using the Roche LC software (Roche, Basel, Switzerland) to determine the threshold cycle (Ct). *BARD1* positive control (HeLa cell line) and PCR negative controls were amplified in each qPCR (quantitative real-time PCR) reaction.

Neuroblastoma samples without *BARD1* expression (*n* = 5) were further investigated toward potential *BARD1* deletion using FISH (*BARD1* specific probe, Empire Genomics; a protocol according to the manufacturer’s guidelines) on the Zeiss microscope AXIO Imager.Z2 in 1000× magnification; image analysis system was MetaSystem (MetaSystems Probes, Heidelberg, Germany).

All inconclusive results were repeated twice. Finally, statistical analysis was performed on 89 neuroblastoma cases and 31 other tumours (GCTs and RMSs), using Statistica software (StatSoft, Kraków, Poland). Paired two-tailed Wilcoxon matched-pairs signed-rank test and two-tailed R Spearman correlation test were applied. Differences between the groups were evaluated with the Mann–Whitney *U* test after checking the normality of the distribution with the Shapiro-Wilk test and the equality of variance with the Levene test.

In all statistical tests, a *p* < 0.05 was considered to be significant. All figures were created in Microsoft Excel. All Supplementary Files heat maps were created by using R language and p heatmap package [36].

## 3. Results

### 3.1. BARD1 in Tumor Tissue

In total, 89 neuroblastoma samples (with three pre- and post-chemotherapy cases), 24 GCTs (19 with paired adjacent tissue), and 7 RMS (all with paired adjacent tissue, and two with samples before and after chemotherapy) passed the quality control and were used for the further analysis.

In the results of *BARD1 FL* expression and the beta isoform for the whole material from neoplastic tissue (*n* = 120; FL: 0.0111 ± 0.0123; β: 0.004 ± 0.0048) and healthy tissue (*n* = 26; FL: 0.0063 ± 0.0048; β: 0.002 ± 0.0025), we found that there were no statistically significant differences in the level of FL expression. There are, however, statistically significant differences (Z = 2.25; *p* = 0.01) between these groups in the level of expression of the beta isoform. In healthy tissue, the beta isoform was expressed at a lower level than in neoplastic tissue.

Comparing the expression in groups of different types of childhood tumours, a higher level of *BARD1 FL* in neuroblastoma (FL: 0.0128 ± 0.0135; β: 0.0045 ± 0.0054) series is noticeable. When compared neuroblastoma group to GCTs (FL: 0.057 ± 0.0055; β: 0.0029 ± 0.0022), in which case expression of *BARD1 FL* is on the lowest level, difference reaches statistical significance (Z = 2.53; *p* = 0.039).

In the case of the beta isoform in neoplastic tissues, statistically, significantly higher expression of the beta isoform was found in a group of ganglioneuroblastoma and ganglioneuroma (FL: 0.0145 ± 0.0136; β: 0.0084 ± 0.0086), comparing to the neuroblastoma group and also in comparison to GCTs (Z = 2.62; *p* = 0.015).

### 3.2. Characteristic of BARD1 Expression in Different Histogenetic Groups of Pediatric Tumors

#### 3.2.1. Neuroblastic Tumors

**Figure 2:**
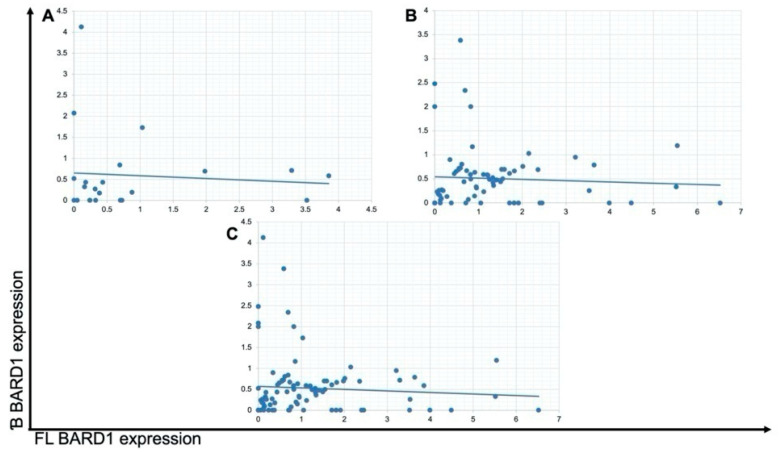
*BARD1 FL* and *BARD1* β isoforms mRNA expression correlation. Neuroblastoma patients were divided into two groups based on the stage of the disease: (**A**) Connected stages 1 & 2 (*n* = 19), (**B**) Stage 3, 4 & 5 (*n* = 70) and (**C**) Patients’ data without stage division (*n* = 89).

**Figure 3:**
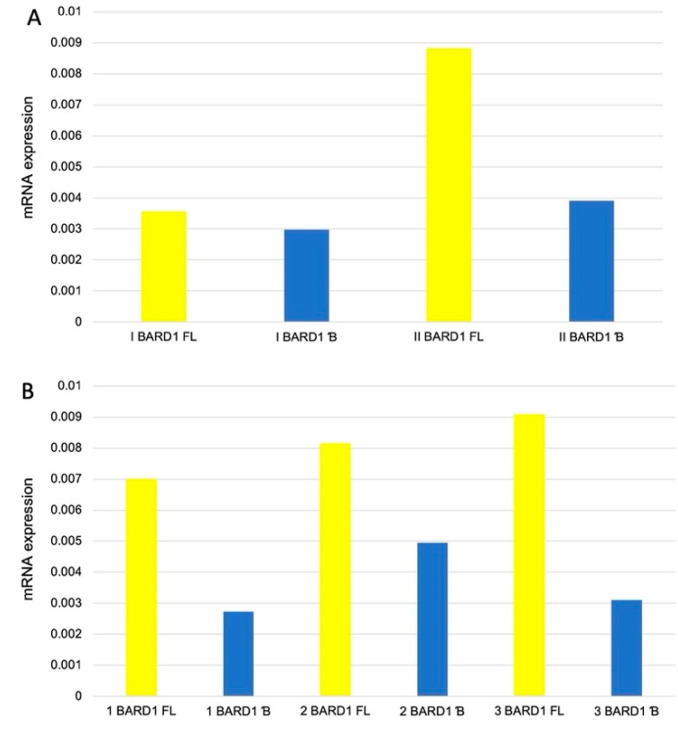
RNA *BARD1* isoforms relative expression of neuroblastoma cases. Neuroblastoma patients were divided into two groups based on the INSS stage (**A**) of the disease and Mitosis-karyorrhexis index (MKI) (**B**). Expression *BARD1* FL and β results were presented for each group. The results are depicted as median.

The majority (*n* = 84/89; 94.4%) of neuroblastoma samples expressed *BARD1 FL*. Interestingly, all five samples that did not express *BARD1 FL* were at stages 3 or 4 and did not either express the *BARD1* β isoform. There was no deletion of the 2q35 region in those samples while checking by FISH. About 73% of the analyzed samples expressed *BARD1* β (*n* = 69/89). However, the group with no *BARD1* β expression is very heterogeneous concerning pathological clinical characteristics. The correlation between *BARD1 FL* and *BARD1*β expression in quantitative analysis in neuroblastoma probes was not found. The results of this analysis are presented in Figure 2. A detailed breakdown analysis was made with access to patho-clinical information: sex, stage, MKI, amplification status, histological subtype, age of diagnosis, localization, histological and clinical risk group. Due to relatively small clinical INSS stage groups, related groups were created: a group I (stage 1 and 2), and group II (stage 3, 4, and 4s). Group II was more numerous, accounting for 79% of cases. In total, lack of *BARD1* β was observed in 27% of samples (*n* = 24/89), 17 in group II (71%, *n* = 17/24) and 7 in group I (29%, *n* = 7/24). Relative expression of *BARD1 FL* and *BARD1* β isoform in INSS grouped data are shown in Figure 3A. Statistically, a significant difference was not observed between NB stage in *BARD1 FL* and *BARD1* β expression analysis. Mitosis-karyorrhexis index (MKI), with the larger number of tumours classified was MKI low (*n* = 40/89; ~45%). Relative expression of *BARD1 FL* and *BARD1* β isoform in MKI subgroups are shown in Figure 3B. In the examined NB group, there was 80% (*n* = 71/89) neuroblastoma type in three histological subtypes: differentiating 32.6%, poorly differentiated 49.3%, and undifferentiated 9.9%. Additionally, 6 (6%) ganglioneuroma cases and 12 (14%) ganglioneuroblastoma were tested. There was a statistically significant difference in beta isoform expression between neuroblastoma (β: 0.0036 ± 0.004) and other histological types (β: 0.0084 ± 0.0086) (Z = 2.57; *p* = 0.01). Higher expression was found in the group consisting of ganglioneuroma and ganglioneuroblastoma compared to different histological subtypes of neuroblastoma. In post-chemotherapy NB tissues, *BARD1 FL* and *BARD1* β isoform expression levels varied, but no pattern was observed. The complex analysis of other factors in different constellations did not reveal any specific patterns.

#### 3.2.2. Germ Cell Tumors

**Figure 4:**
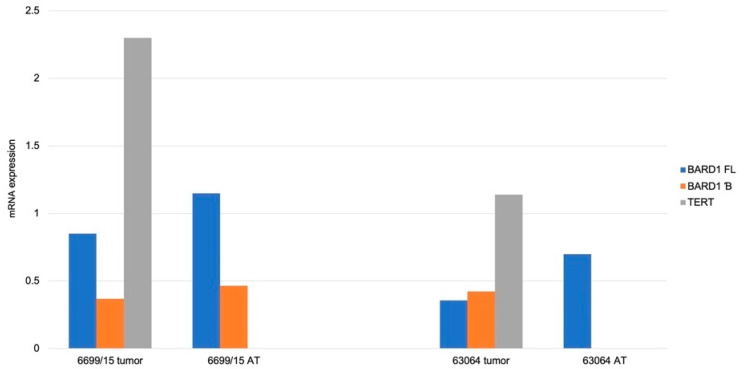
*TERT* and *BARD1* isoforms expression level of yolk sac representative samples. Histogram of mRNA expression for *TERT* and *BARD1* isoforms of representative samples. Results are depicted as median. AT—adjacent tissue.

**Figure 5:**
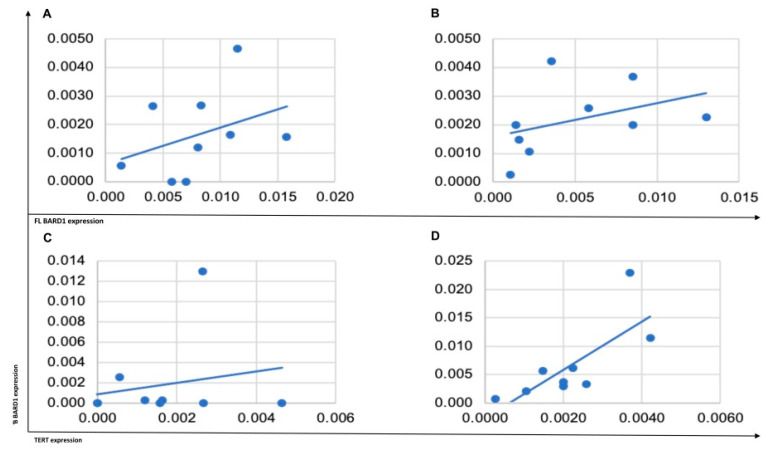
*TERT*, *BARD1 FL*, and β isoform mRNA expression correlation in yolk sac samples. (**A**) Correlation between *BARD1 FL* and β isoform in unchanged histological tissue. (**B**) Correlation between *BARD1 FL* and β isoform in tumor tissue. (**C**) Correlation between *BARD1* β isoform and *TERT* in unchanged histological tissue. (**D**) Correlation between *BARD1*β isoform and *TERT* in tumor tissue.

All analyzed GCTs expressed *BARD1 FL*, with statistically significant differences between the *BARD1 FL* expression in the tumour (0.005 ± 0.004) and adjacent tissue (0.008 ± 0.0042) in the yolk sac tumour (YCT) (Z = 2.19; *p* = 0.024). Higher expression was noticed in adjacent tissue. While expression of *BARD1* β was detected in 23 tumor tissues (*n* = 23/24; 96%) and 16 adjacent tissues (*n* = 16/19; 84%). *TERT* was not expressed in 52% (*n* = 10/19) of the adjacent tissues and only in 8% (*n* = 2/24) of tumour tissues (teratomas). Interestingly, when analyzing *BARD1* β compared to *FL* expression depending on GCTs subtype, we found a higher correlation in YCT tissue (0.617 ± 0.151) than adjacent healthy tissues (0.243 ± 0.0729) (Z = 2.07; *p* = 0.02). However, such differences were not found in teratoma and dysgerminoma (expression level for immature teratoma samples are presented in Supplementary File S2). Data for representative samples are presented in Figure 4 and for all yolk sac-in Supplementary File S3. Correlation data and analysis for yolk sac tumours are presented in Figure 5. Moreover, YCT showed a higher expression of *BARD1* β and *TERT*. However, only in tumor samples statistically significant positive correlation was found between *BARD1* β and *TERT* expression(*r* = 0.8824; *p* = 0.003).

#### 3.2.3. Rhabdomyosarcoma

**Figure 6:**
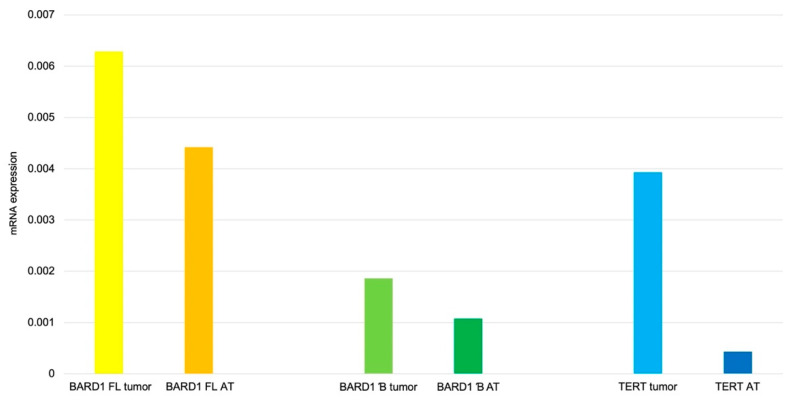
*TERT* and *BARD1* isoforms expression levels of RMS samples. Histogram of mRNA expression for *TERT, BARD1 FL*, and *BARD1* β isoform of RMS samples. Results are depicted as median. AT-adjacent tissue.

All analyzed pilot group RMS tumour tissue expressed *BARD1 FL* and *BARD1* β (*n* = 7/7; 100%), while in adjacent tissue, one did not express *BARD1 FL*, and two did not express *BARD1* β (*n* = 5/7; 71%). *TERT* was detected in 6 tumor (86%) and 4 (57%) adjacent tissue. We found higher expression of *BARD1* β isoform in neoplastic tissue (0.0032 ± 0.00095) than adjacent healthy tissues (0.00067 ± 0.000223) (Z = 2.36; *p* = 0.018). A statistically significant difference was also performed between tumour (0.00576 ± 0.00164) and healthy tissue (0.00079 ± 0.000385) in *TERT* expression (Z = 2.19; *p* = 0.028). Statistically significant results for RMS probes are presented in Figure 6.

In the RMS group, there were 2 cases with samples collected after chemotherapy treatment. The expression of *BARD1 FL*, *BARD1* β, and *TERT* decreased after treatment in these samples.

## 4. Discussion

In cancer cells, the mRNA assembly process, like other processes, undergoes specific changes that may result in disease progression. In a healthy cell, naturally occurring isoforms are in the right proportions, allowing harmonic and carefully controlled operation of all processes. The alternative assembly processes may also result in new, unfavourable, isoforms [37].

*BARD1* is an example of a gene whose changes can lead to cancerogenesis. The full-length BARD1 is classified as a tumour suppressor and the main partner of BRCA1 (forming a heterodimer via the RING domain). beta is one of the most studied isoforms; lack of RING domain makes its incapable of interacting with BRCA1. *BARD1* alternative isoforms have been described, among others, in the colorectal, lung, gynaecological cancers, and neuroblastoma [38,39]. Here, we describe the analysis of *BARD1* β in three types of childhood cancers: neuroblastoma, germ cell tumours, and rhabdomyosarcoma. The previous data suggest that a high level of *BARD1* β is associated with an adverse prognosis in different cancer types [5,12,40,41].

In our cohort, we confirmed a higher expression of *BARD1* β in the tumour but not in adjacent tissue; however, we did not observe any difference in *BARD1 FL* between cancer and non-neoplastic control. This observation contradicts Cimmino et al., who reported a reduction of *BARD1 FL* in cancer cells [6]. A possible explanation of this phenomena is a limitation of the TaqMan probe that we used in this study. The Hs00957649_m1 binds on exon 3 and 4 junction, therefore will also detect other isoforms that have these exons.

BARD1 is also an important factor involved in all stages of spermatogenesis and embryogenesis. Its isoforms were found in the human cytotrophoblast [42], and depletion of *BARD1* leads to lethality in embryonic life or development or cerebral malformations [7]. Besides, the BRCA1-BARD1 complex plays an essential role in meiosis, homologous recombination, and also takes part in brain and neural crest line morphogenesis [43]. Therefore, we hypothesise that *BARD1* alternative isoforms might play a role in most childhood tumours and as such, these cancers might share similar *BARD1* profiles.

The genome-wide association study (GWAS) associated several *BARD1* variants with susceptibility to high-risk neuroblastoma [44], mainly rs6435862 T>G was related with overexpression of *BARD1* β [5]. Additional studies, genotyping neuroblastoma patients, were conducted in other populations [45,46,47]; however, not all of them confirmed previous findings [45]. Our cohort of patients has not been genotyped prior to *BARD1* expression analysis; therefore, our study was performed in an unselected cohort, limiting possible bias of earlier molecular results.

Earlier studies demonstrated that BARD1 β blocks the apoptosis of neuroblastoma cells and stabilizes the Aurora kinase A and B, which are essential players in NB biology [5,7]. However, our results do not demonstrate a correlation between expression of *BARD1* β and patho-clinical features in neuroblastoma and lack of *BARD1* β (but not *BARD1 FL*) did not correlate with more favourable phenotype. This observation was unexpected, especially given the direct impact of *BARD1* β in cancerogenesis.

Furthermore, we observed a statistically significant difference in *BARD1* β expression in different histological subtypes; with *BARD1* β being highly expressed in more mature ganglioneuroma/ganglioneuroblastoma (*p* = 0.01). These NB family members are more mature and characterized with a higher degree of neural cells differentiation, and significant content of Schwannian stroma [48], and have a better prognosis than neuroblastoma. A possible explanation is a link between neural crest differentiation, maturing morphology in NB tumours and a role of *BARD1* isoforms in these processes; however, this phenomenon needs further evaluation.

In our cohort, we identified five tumours without *BARD1* (both β and FL) expression, to assess *BARD1* gene status we performed FISH analysis. However, the result did not confirm a potential deletion of *BARD1*. Therefore, the absence of *BARD1* expression may indicate other inactivating mechanisms, like promotor methylation or genetic mutation [7,49]. Interestingly, the absence of *BARD1* due to promoter methylation was observed only once, in a series of colon cancers [13].

Importantly, here we report for the first time the expression of *BARD1* in other childhood cancers, including germ cell tumours (GCTs) and rhabdomyosarcoma (RMS). However, due to a low number of RMS cases, this analysis should be considered as a pilot study, which findings require further evaluation. In GCTs and RMS cohorts, we additionally assessed the *TERT* expression level, as it was previously reported that BARD1 plays a role in telomere stability [11,40,50]. Therefore, we wanted to explore a possible correlation between *BARD1* expression and *TERT* level.

Teratomas and yolk sac tumours histologically represent the tumors with a wide pattern of differentiation–the first contains mixture of elements of three germ layers, since the second recapitulates specific endodermal derived structures [51,52]. Dysgerminoma is built of the undifferentiated primordial- like germ cells with lymphocytic stroma [53]. Therefore, our hypothesis assumed that BARD1 taking part in early stage of development plays an essential role in the pathology of these specific neoplasms. GCTs develop mainly in gonads, where migrating primordial germ cells reach their destination during embryogenesis, and most of GCTs occur in males, which rises another link with BARD1 being naturally elevated in testis [54].

In our study group, *BARD1* β was higher expressed in a tumour that non-neoplastic adjacent tissue, with the difference being statistically significant in yolk sack tumours. Besides, there was a positive correlation in expression of *BARD1* β and *TERT,* which support the known role of *BARD1* β in cancerogenesis. Interestingly, this correlation was not present in other analysed GCTs subtypes (immature teratoma and dysgerminoma). Nevertheless, it might be partially explained by variegated histological characteristics of these neoplasms.

Lastly, we examined the expression of *BARD1* (FL and β) in a small group of aggressive soft tissue sarcoma, RMS. The *TP53, NF1*, *NRAS, CDK4, MYCN, GLI, MDM2, FGFR1,* and *FGFR4* are listed among the most frequently altered in these tumours, interestingly they play a role in the cell cycle regulation and DNA repair, and interact with BARD1 [55,56]. Also, chromosome 2 alterations (where *BARD1* is located, 2q34-35) are commonly found in RMS [57]. Our research showed that *BARD1*β isoform and the *TERT* gene were significantly more strongly expressed in the neoplastic tissue compared to the healthy control tissue.

Additionally, in two RMSs we had access to the material pre- and post-chemotherapy, where expression of *BARD1* β was dramatically reduced post-treatment (accompanied with *TERT* being below detection limits). In our opinion, although this observation has a preliminary character (due to the limited size of the studied group), it designates the role of BARD1 β not only in RMS pathobiology but also carcinogenesis itself.

It is worth emphasizing that the study conducted requires further confirmation using other methods, larger groups of respondents of different ethnic origins and comparison with available databases. Validation of the obtained results by using methods with other strengths and weaknesses would avoid limitations resulting from the homogeneity of the analytical workshop applied.

To summarise, here we examine the role of BARD1 β in a series of neuroblastoma tumours, but also in a relatively poorly recognized GCTs and RMS. Our results indicated a highly probable function of BARD1 and its isoforms in other childhood tumours’ pathobiology; however, it needs to be verified in a larger cohort of patients.

## 5. Conclusions

In our work, we confirmed the higher expression of *BARD1* β isoform in neoplastic tissues of pediatric tumors. Here, we show that there may be a specific *BARD1* isoforms pattern in germ cell tumors, rhabdomyosarcoma, and neuroblastoma subtypes. The differences in *BARD1* expression depend on the histological type of neoplasm, and the level of maturation in neuroblastic tumours. Our findings confirm the association with oncogenesis of *BARD1* β not only in the neuroblastoma, but for the first time also in selected GCT and RMS. 

## Figures and Tables

**Table 1:** Patho-clinical characteristics of tumours–neuroblastoma analyzed cases.

Age (range in months (median))	1–169 (30)
Sex	
boys	48 (54%)
girls	41 (46%)
Stage (INSS)	
1	7 (8%)
2	12 (13%)
3	30 (34%)
4	33(37%)
4s	7 (8%)
Mitosis Karyorrhexis Index (MKI)	
low	40 (45%)
intermediate	25 (28%)
high	24 (27%)
Histological category	
Neuroblastoma	71 (80%)
undifferentiated	7 (9%)
poorly differentiated	35 (50%)
differentiating	29 (41%)
Ganglioneuroblastoma	12 (13%)
Ganglioneuroma	6 (7%)
Tumor localization	
adrenal	46 (51%)
extra-adrenal	43 (49%)
*NMYC* status	
amplification	18 (20%)
no amplification	70 (79%)
unknown	1 (1%)
Histological risk group (INPC)	
favorable	51 (57%)
unfavorable	38 (43%)
Clinical risk group	
low	26 (29%)
intermediate	27 (30%)
high	36 (41%)

**Table 2:** Patho-clinical characteristics of germ cell tumors.

Histological Type	Group Characteristic:		
	Age (Range in Months (Median))	Sex (Boys/Girls)	Tumor Location
Immature Teratoma (7)	Congenital-204 (55)	4 (57%)/3 (43%)	Testicle (3) (43%), Mediastinum (1) (14%), Sacro-caudal area (3) (43%)
Yolk sac tumor (9)	6–138 (51)	8 (89%)/1 (11%)	Testicle (8) (89%), Ovary (1) (11%)
Dysgerminoma (10)	84–204 (156)	10 girls (100%)	Ovary (10) (100%)

**Table 3:** Patho-clinical characteristics of Rhabdomyosarcoma.

Age (Range in Months (Median))	36–156 (104.6)
Sex	
boys	5 (71%)
girls	2 (29%)
Tumor localization	
Thorax, head and neck	2 (29%)
Limbs	3 (42%)
Pelvic	2 (29%)
Histological category	
RMS alveolare	3 (43%)
RMS embryonale	4 (57%)

## Data Availability

Raw data, detailed protocols, and analyzed data during this study are available from the corresponding author on reasonable request.

## Supplementary Materials

The following are available online at https://www.mdpi.com/2073-4425/12/2/168/s1. File S1: Representative photos of analyzed neoplasms, File S2, BARD1FL and beta isoforms expression level in neuroblastoma sample, File S3, BARD1isoforms and TERT expression level of all teratomas samples.
