# Supplementary material for: Expression of BARD1 β Isoform in Selected Pediatric Tumors
Source: Genes (Basel). 2021 Jan 26;12(2):168. doi: 10.3390/genes12020168 (PMC7911681; doi:10.3390/genes12020168)

Supplementary Files

Expression of BARD1 beta isoform in selected pediatric tumors.

Anna Jasiak[1,2], Natalia Krawczynska[3,1], Mariola Iliszko[1,2], Katarzyna Czarnota[4], Kamil Buczkowski[4,5], Joanna Stefanowicz[6], Elzbieta Adamkiewicz-Drozynska[6], Grzegorz Cichosz[1], Ewa Izycka-Swieszewska[4,5]*

[1]Department of Biology and Medical Genetics, Medical University of Gdansk, 1 Debinki St. 80-211 Gdansk, Poland
[2]Laboratory of Clinical Genetics, University Clinical Centre, 17 Smoluchowskiego St. 80-210 Gdansk, Poland
[3]Department of Molecular and Integrative Physiology, the University of Illinois at Urbana-Champaign, 407S Goodwin Ave, Urbana, IL, 61801, USA
[4]Department of Pathology and Neuropathology, Medical University of Gdansk, 1-6 Nowe Ogrody St. 80-803 Gdansk, Poland
[5]Department of Pathomorphology Copernicus Hospitals, 1-6 Nowe Ogrody St. 80-803 Gdansk, Poland
[6]Department of Pediatrics, Hematology, Oncology, Medical University of Gdansk, 1 Debinki St. 80-211 Gdansk, Poland

* Author to whom correspondence should be addressed.
*eczis@gumed.edu.pl

Supplementary File 1. Representative photos of analyzed neoplasms.

Hematoxylin and eosin staining of: neuroblastoma subtypes A- poorly differentiated (400x), B – undifferentiated (600x), C – differentiating (400x), D – ganglioneuroblastoma (200x), E – ganglioneuroma (400x), F, G – dysgerminoma (400x), H – yolk sac tumor (400x), I, J- teratoma (200x), K – alveolar rhabdomyosarcoma (200x), L – embryonal rhabdomyosarcoma (400x).

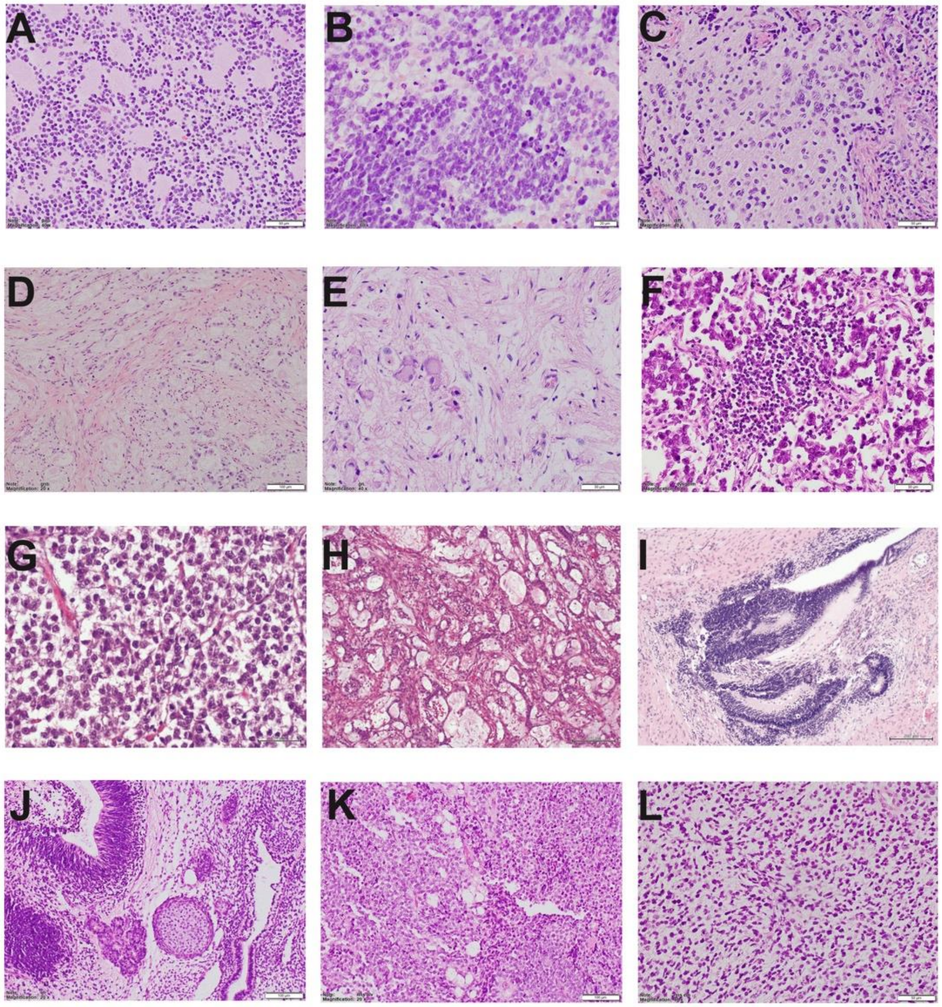

Supplementary File 2. ***BARD1*** **FL and beta isoforms expression level in neuroblastoma samples.**

A - no clustered heatmap for tested genes mRNA expression with division based on INSS scale (International Neuroblastoma Staging System Criteria); **B** - clustered heatmap.

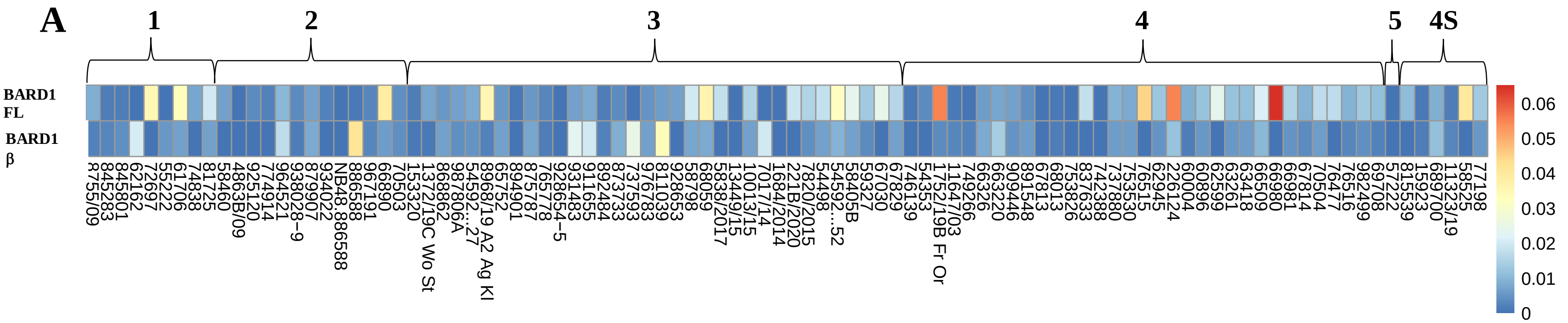

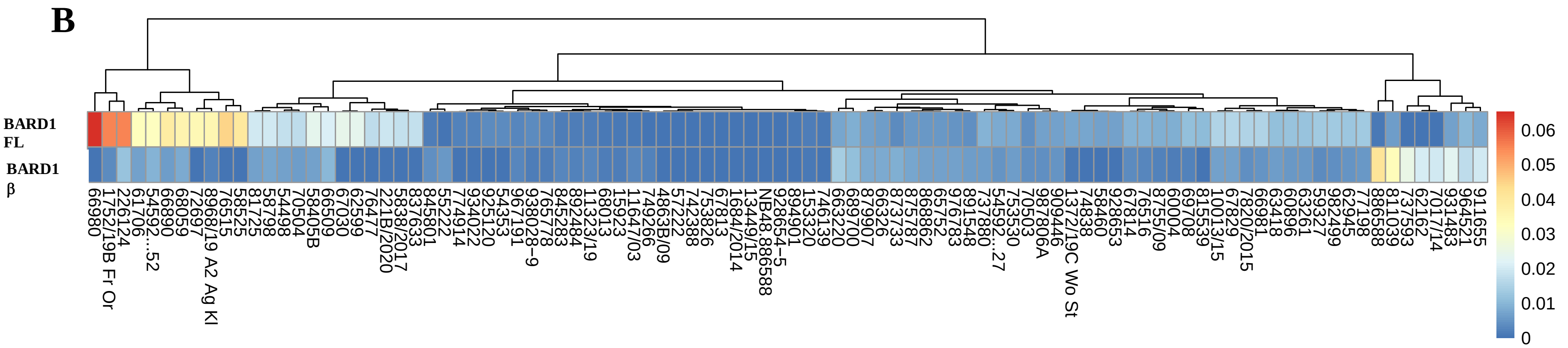

Supplementary File 3. ***BARD1*** **isoforms and** ***TERT*** **expression level of all teratomas samples.**
A – no clustered heatmap for tested genes mRNA expression. **B** – clustered heatmap. AT – adjacent tissue; NE – no expression.

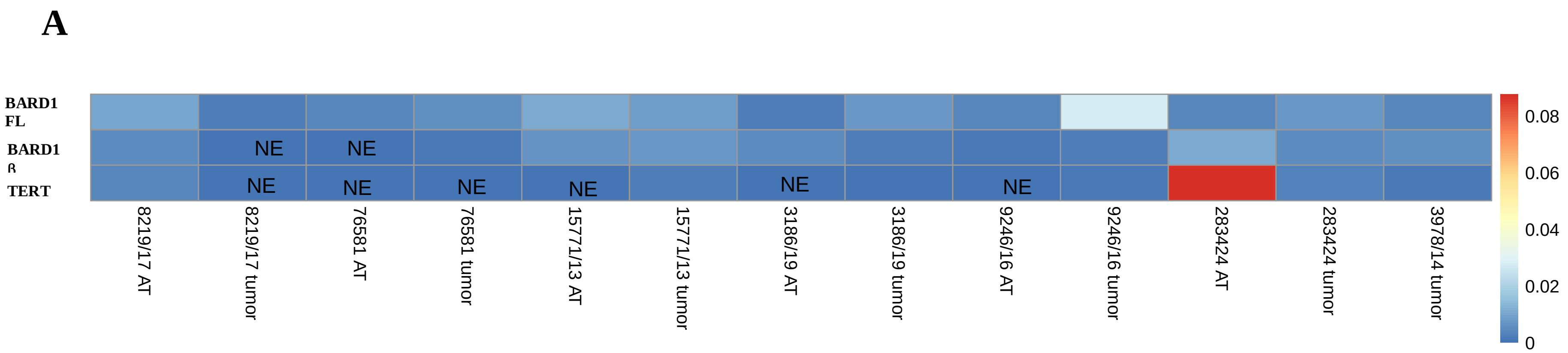

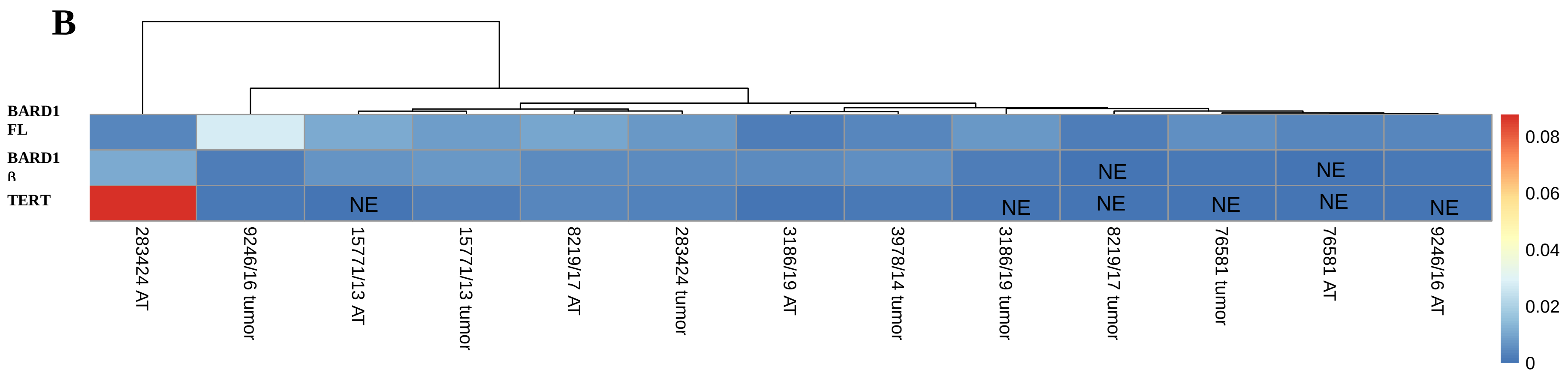

Supplementary File 4. *BARD1* isoforms and *TERT* expression level of all yolk sack samples.

No clustered (**A**) and clustered (**B**) heatmaps for tested genes mRNA expression of all tested samples (tumorous and adjacent tissue – AT). NE – no expression.

A

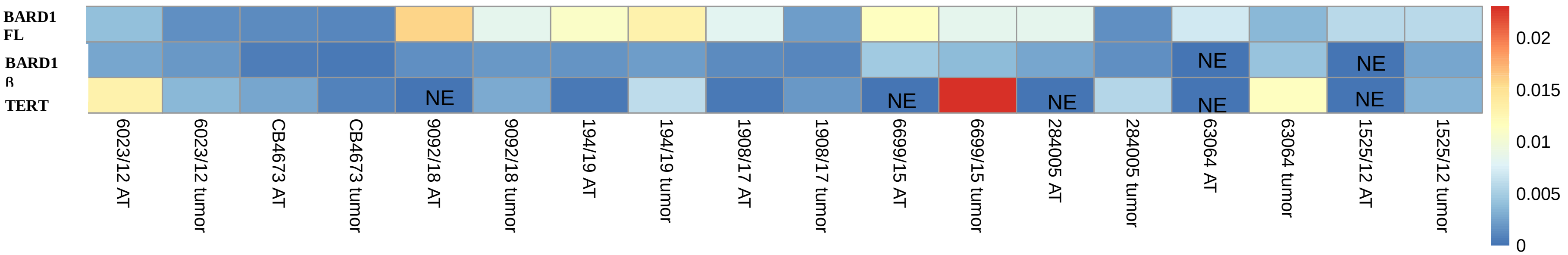

B

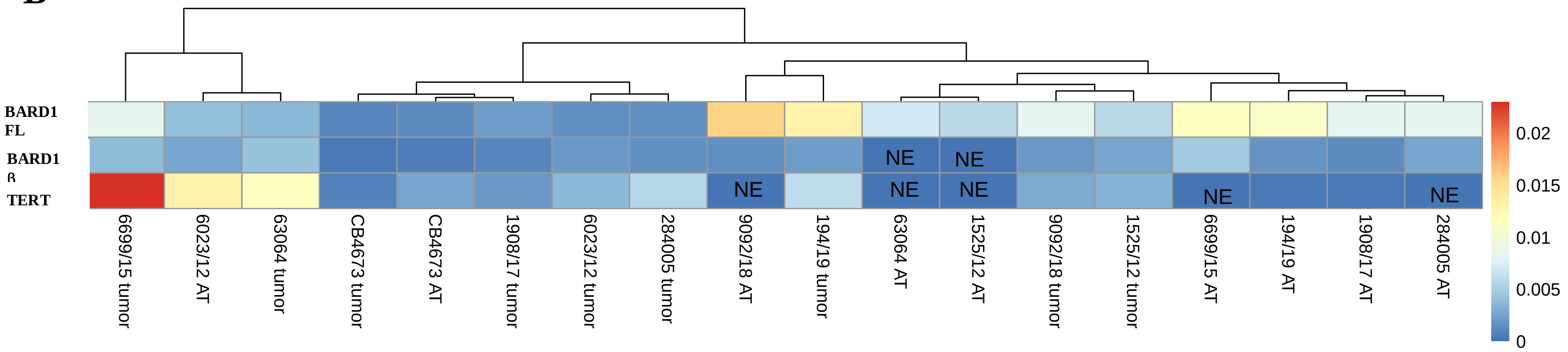

Supplement: Supplementary file 1 [file genes-12-00168-s001.pdf]
